# Case Report: A rare solitary peroneus longus metastasis from rectal cancer managed with multimodal therapy

**DOI:** 10.3389/fmed.2026.1744724

**Published:** 2026-02-25

**Authors:** Qiangming Liao, Junjun Yan

**Affiliations:** 1 Department of Gastrointestinal Surgery, Jiujiang City Key Laboratory of Cell Therapy, Jiujiang No.1 People’s Hospital, Jiujiang, China; 2 Department of Gastroenterology Surgery, The First Affiliated Hospital, Jiangxi Medical College, Nanchang University, Nanchang, China; 3 Department of Gastroenterology, Jiujiang City Key Laboratory of Cell Therapy, Jiujiang No.1 People’s Hospital, Jiujiang, China

**Keywords:** case report, disease-free survival, multimodal therapy, rectal cancer, solitary peroneus longus metastasis

## Abstract

Solitary skeletal muscle metastasis from rectal cancer is exceptionally rare and carries a generally poor prognosis, with no established treatment guidelines currently available. We present a case of a patient with advanced rectal cancer (T3cN2bM0) who refused recommended neoadjuvant chemoradiotherapy and underwent laparoscopic radical resection alone. Subsequently, the patient developed an isolated metastasis to the left peroneus longus muscle merely three months postoperatively. The metastasis presented as a painful mass, and ^18^F-Fluorodeoxyglucose positron emission tomography/ computed tomography (^18^F-FDG PET/CT) revealed its hypermetabolic nature, suggesting malignancy. This suspicion was confirmed histopathological, establishing the diagnosis of skeletal muscle metastasis from rectal adenocarcinoma. Following a combined treatment of surgical resection and systemic chemotherapy, the patient remarkably remained free of local recurrence or further metastasis throughout a 30-month follow-up period. This case underscores the importance of clinical alertness for rare skeletal muscle metastases and highlights that individualized treatment is essential for improving patient prognosis.

## Introduction

Colorectal cancer ranks third among all cancers worldwide, accounting for 9.6% of cases, and it is the second leading cause of cancer-related deaths, with a proportion of 9.3% (1). Metastasis of colorectal cancer occurs via lymphatic, hematogenous, and direct spread pathways. Approximately 20% of colorectal cancer cases are found to have metastases at initial evaluation, with the most common distant metastatic sites being the liver, lungs, peritoneum, bones, and extra-regional lymph nodes (2). In contrast, intramuscular metastases are notably uncommon, comprising less than 1% of all metastatic lesions (3). However, the present case contributes valuable insights to this limited body of literature by demonstrating that prolonged disease-free survival can be achieved through aggressive multimodal therapy, even in a high-risk scenario characterized by an early, isolated recurrence (4–8). This case not only highlights the critical importance of individualized patient management but also challenges conventional prognostic assumptions associated with such rare oligometastatic presentations.

## Case report

A 55-year-old male presented with a 6-month history of altered bowel habits, characterized by hematochezia and increased defecation frequency, in the absence of systemic symptoms such as weight loss or abdominal pain. He had no family history of gastrointestinal malignancies. Physical examination revealed no bilaterally enlarged superficial lymph nodes, and no tenderness was noted. Digital rectal examination demonstrated smooth rectal mucosa and no obvious palpable lesions or nodules; nevertheless, blood was noted on withdrawal of the finger. Consequently, a colonoscopy was performed, which identified a rectal mass 8 cm from the anal verge. The subsequent histopathological examination of a biopsy confirmed the diagnosis of moderately differentiated adenocarcinoma.

**Figure 1:**
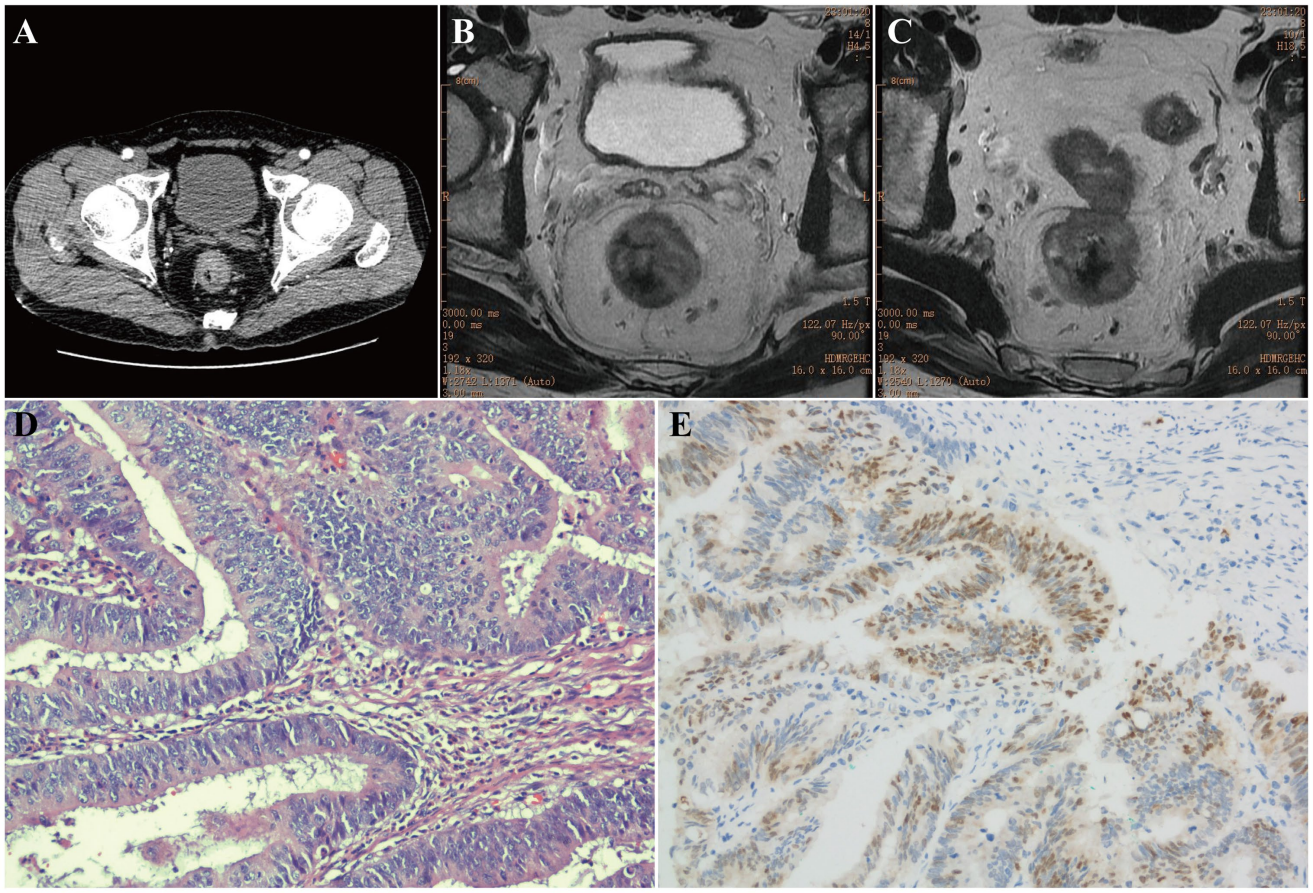
Radiologic and pathologic features of the primary rectal adenocarcinoma. **(A)** Axial contrast-enhanced computed tomography (CE-CT) image revealed a heterogeneously enhancing mass in the mid-to-lower rectum. **(B)** Axial T2-weighted magnetic resonance imaging (MRI) confirmed a cT3c lesion with 5–15 mm extramural extension into the perirectal fat. **(C)** Axial T2-weighted MRI identified four suspicious regional lymph nodes, consistent with cN2b disease. **(D)** Histopathological examination (H&E, ×200) demonstrated moderately differentiated adenocarcinoma. **(E)** Immunohistochemical staining (IHC, ×200) for CDX-2 was positive, confirming intestinal-type rectal adenocarcinoma.

Pretherapeutic staging commenced with contrast-enhanced computed tomography (CE-CT) of the chest, abdomen, and pelvis. The scan revealed a heterogeneously enhancing mass in the mid-to-lower rectum (Figure 1A), with no radiological evidence of distant metastasis (cM0). For precise local staging, pelvic magnetic resonance imaging (MRI) was subsequently performed. The MRI confirmed a cT3c lesion, demonstrating 5–15 mm of extramural extension into the perirectal fat (Figure 1B), and identified four suspicious regional lymph nodes, establishing a cN2b status (Figure 1C). These collective findings defined the pre-treatment clinical stage as cT3cN2bM0. In accordance with this stage, neoadjuvant chemoradiotherapy (nCRT) was recommended to optimize local control; however, the patient declined. He subsequently underwent laparoscopic radical rectal cancer resection with prophylactic ileostomy in October 2022. Histopathological examination of the resected specimen confirmed moderately differentiated adenocarcinoma (Figures 1D–E) and revealed lymphovascular invasion along with metastasis in two of fourteen pericolic lymph nodes, downstaging the disease to pT3N1bM0.

Given the high-risk pathological features, adjuvant therapy was initiated to reduce the risk of recurrence of disease. The treatment regimen commenced with two cycles of XELOX (capecitabine and oxaliplatin) between November and December 2022. Subsequently, the patient underwent concurrent chemoradiotherapy from December 2022 to January 2023, which targeted the pelvic surgical bed and regional lymph nodes, using oral capecitabine as a radiosensitizer. The adjuvant course was completed with a third consolidative cycle of XELOX in February 2023.

**Figure 2:**
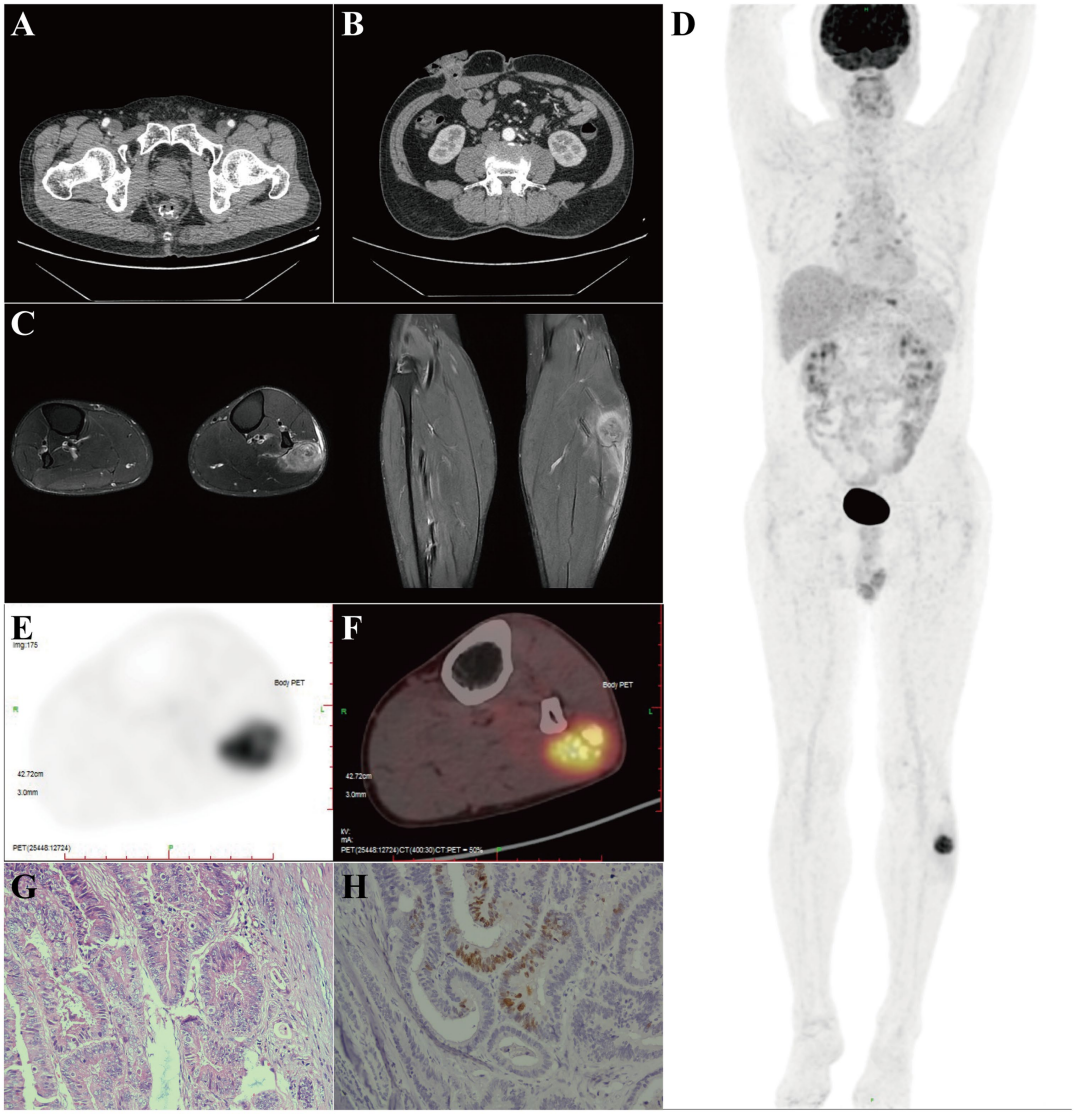
Detection and confirmation of a solitary calf metastasis. **(A,B)** Axial CE-CT of the pelvis **(A)** and abdomen **(B)** showed no significant abnormalities during treatment follow-up. **(C)** Axial and coronal T2-weighted/short-tau inversion recovery (STIR) MRI of the left calf demonstrated a poorly marginated, invasive mass. **(D)** Whole-body ^18^F-Fluorodeoxyglucose positron emission tomography (^18^F-FDG PET) maximum intensity projection (MIP) image revealed a solitary focus in the left lower limb. **(E,F)** Standardized uptake value PET **(E)** and fused PET-CT **(F)** images confirmed a hypermetabolic lesion in the peroneus longus muscle (SUVmax 9.4). **(G,H)** Histopathological examination of the resected specimen confirmed metastatic intestinal-type adenocarcinoma, as shown by **(G)** hematoxylin and eosin (H&E) staining (×200) and **(H)** positive nuclear immunohistochemical (IHC) staining for CDX2 (×200), consistent with origin from the primary rectal tumor.

Subsequent CE-CT of the abdomen and pelvic showed no significant abnormalities, with treatment progressing uneventfully (Figures 2A,B). Nevertheless, the patient presented with a new complaint of left lower limb pain, which further complicated the clinical assessment. Physical examination revealed a firm, fixed 3 cm × 2 cm mass in the left mid-upper calf. MRI of the leg demonstrated a poorly marginated intramuscular mass in the proximal lateral fibular region. The lesion appeared hypointense on T1-weighted images, heterogeneously hyperintense on T2-weighted/STIR sequences, demonstrated diffusion restriction, and exhibited features of local invasion, including muscle bundle destruction and obliteration of intermuscular septa (Figure 2C). These combined radiological features raised suspicion for a metastatic lesion. Therefore, to definitively characterize the suspicious calf lesion and thereby complete systemic staging, ^18^F-Fluorodeoxyglucose positron emission tomography/CT (^18^F-FDG PET/CT) was performed. It confirmed a hypermetabolic focus in the peroneus longus muscle (SUVmax 9.4), findings diagnostic of metastasis (Figures 2D–F). In February 2023, the patient underwent concurrent ileostomy reversal and complete resection of the limb mass. Histopathological analysis confirmed the diagnosis of metastatic intestinal-type adenocarcinoma, consistent with the primary rectal tumor (Figures 2G,H). This presentation represents an unusual site of dissemination, given the rarity of skeletal muscle metastases from colorectal cancer.

In response to this metastatic progression, the systemic therapy regimen was adjusted to incorporate bevacizumab in combination with irinotecan and capecitabine. One cycle was administered in March 2023; however, the treatment was poorly tolerated, and the patient subsequently declined further chemotherapy. Despite this, at the 30-month follow-up, the patient exhibited clinical stability with no radiological evidence of local recurrence or distant metastasis. This outcome underscores the potential for favorable long-term control in complex, metastatic colorectal cancer through a tailored, multimodal strategy that strategically integrates surgery, systemic therapy, radiotherapy, and persistent surveillance.

## Discussion

Skeletal muscle metastases (SMMs) from colorectal cancer are uncommon, with isolated involvement of the peroneus longus muscle representing an exceptionally rare clinical scenario. Here, we report the case of a 55-year-old man with locally advanced rectal adenocarcinoma who developed an isolated peroneus longus metastasis within a short disease-free interval of only three months after declining neoadjuvant chemoradiation and undergoing curative resection. Although such an early, this patient achieved long-term disease-free survival through multimodal treatment. This case highlights both the clinical challenges in managing rare metastatic presentations and the potential for favorable outcomes even in high-risk scenarios, thereby challenging conventional prognostic assumptions.

As the largest organ system in the human body, skeletal muscle is nevertheless an infrequent site of metastasis from solid tumors (9). This resistance is attributed to mechanical barriers such as fascial sheaths, as well as a biochemical microenvironment that is generally non-conducive to tumor cell colonization (10, 11). Most SMMs occur in the context of disseminated disease, and isolated intramuscular metastasis is often considered an indicator of systemic recurrence, typically portending an unfavorable prognosis (11–13). In colorectal cancer, although isolated SMMs have been sporadically documented, they are more frequently observed in regional drainage areas (such as the gluteal region or psoas muscle) or in highly vascularized large muscle groups of the lower limbs (5, 7, 14). Isolated metastases to the distal muscle groups of the lower limbs, however, are exceptionally rare, with only three documented cases in the literature to date (7, 15, 16). The interval between primary tumor resection and the detection of isolated muscular metastasis is highly variable (4, 7, 8, 15–20). In the present case, this interval was remarkably short, at merely three months. Notably, the emergence of isolated muscle metastases within such a brief period following curative surgery is an exceedingly rare occurrence and is typically associated with a grave prognosis. Contrary to this conventional expectation, our patient achieved long-term survival following systematic therapy. This outcome underscores the pivotal role of multidisciplinary intervention and individualized management in altering the disease course and improving prognosis, even in high-risk scenarios.

This case underscores that in patients with a history of malignancy, a newly developed soft-tissue mass should be prioritized as a potential metastasis (11, 21, 22). Although the differential diagnosis for SMMs includes primary soft-tissue sarcoma and benign entities such as abscess or hematoma (12, 23, 24), the absence of local inflammatory signs in this patient made an infectious process unlikely. MRI is the preferred modality for evaluating soft-tissue masses, as it optimally delineates local anatomical relationships and invasive features (13). One of the most suggestive MRI characteristics of SMMs is the presence of significant peritumoral edema and an infiltrative growth pattern, which contrasts with the relatively well-defined margins often seen in many primary soft-tissue sarcomas. In the present case, MRI clearly demonstrated destruction of muscle bundles and intermuscular septa, features more consistent with the aggressive behavior of a metastatic lesion (Figure 2C). ^18^F-FDG PET/CT further supported the malignant nature of the lesion through its high metabolic activity and, crucially, confirmed the oligometastatic status by ruling out other distant foci (Figures 2D–F). Ultimately, the diagnosis was confirmed, with the biopsy revealing an intestinal-type adenocarcinoma consistent with the patient’s known primary tumor.

Currently, there are no standardized therapeutic guidelines for isolated SMMs, and its management is intricately linked to patient prognosis (14, 25, 26). The favorable outcome achieved in our patient can be largely attributed to the decisive, multimodal treatment approach adopted. This approach prioritized aggressive local control through complete surgical resection of the isolated metastasis (14), integrated with systemic therapy. Systemic treatment selection followed principles for high-risk colorectal cancer. Initial adjuvant XELOX was aligned with guideline recommendations based on the primary tumor’s high-risk features (pT3N1b) (27). Upon metastatic progression during this regimen, the patient was transitioned to a regimen of capecitabine, irinotecan, and bevacizumab—a decision considering the patient’s decline of genetic testing and limited healthcare access and supported by evidence for anti-angiogenic therapy in metastatic disease, particularly given the short disease-free interval (27). In contrast, when complete resection is not feasible due to technical constraints, the patient’s comorbidities, or the presence of disseminated disease, the prognosis associated with palliative radiotherapy and/or chemotherapy is generally poor (23, 28). This experience reinforces the potential of a curative-intent, multimodal approach to achieve long-term survival in carefully selected patients with isolated metastatic recurrence.

In conclusion, this case offers several key lessons for clinical practice. First, any new soft tissue mass in a cancer patient, regardless of how uncommon the location, must be rigorously investigated for potential metastasis. Second, for isolated SMMs, a comprehensive, multidisciplinary evaluation followed by an aggressive treatment strategy combining complete surgical resection with systemic therapy can yield excellent long-term results. This case serves as a valuable paradigm for managing such rare metastatic presentations. This study has limitations inherent to a single case report, and the findings are not generalizable. Furthermore, the lack of molecular profiling precludes a deeper understanding of the tumor biology underlying this solitary metastasis and its treatment response, highlighting the need for future collaborative, biomarker-integrated studies.

## Data Availability

The original contributions presented in the study are included in the article/supplementary material, further inquiries can be directed to the corresponding author.
